# Study on Rapeseed Albumin Hydrolysis by PrtS Protease from *Streptococcus thermophilus* and Bioactivity Characterization of Resulting Hydrolysates

**DOI:** 10.3390/foods14132235

**Published:** 2025-06-25

**Authors:** Zeeshan Hafeez, Sophie Beaubier, Arnaud Aymes, Ségolène Christophe, Samina Akbar, Romain Kapel, Laurent Miclo

**Affiliations:** Université de Lorraine, CNRS, LRGP, F-54000 Nancy, France

**Keywords:** rapeseed albumins, enzymatic hydrolysis, PrtS protease, *Streptococcus thermophilus*, lactic acid bacteria, bioactivity

## Abstract

Lactic acid bacteria are well known for hydrolyzing milk proteins, but their application to plant proteins remains limited. This study evaluated the ability of the cell-wall-anchored PrtS protease from two *Streptococcus thermophilus* strains to hydrolyze rapeseed albumins (RAs), aiming to generate bioactive peptides with potential food functionality. The specific activity of PrtS was first determined using a chromogenic substrate. RAs were then hydrolyzed using 10X- and 100X-concentrated cell pellets of each strain to assess the hydrolysis kinetics and the enzymatic mechanism. The results showed concentration-dependent hydrolysis, with protein conversion and the degree of hydrolysis increasing threefold at 100X for both strains. Despite the increased hydrolysis, the peptides produced had similar average sizes, averaging at five amino acids, indicating a consistent “one-by-one” cleavage mechanism. The in vitro testing of the RA hydrolysates produced with 100X PrtS from *S. thermophilus* LMD-9 revealed dose-dependent antioxidant activity comparable to native RAs. Importantly, unlike native RAs, these hydrolysates did not induce increased secretion of the pro-inflammatory mediator IL-8 in inflamed HT-29 cells, suggesting a reduced pro-inflammatory potential. These findings demonstrate that PrtS protease from *S. thermophilus* can effectively hydrolyze rapeseed proteins to produce functional hydrolysates with improved bioactivity profiles. Such hydrolysates have promising applications as functional ingredients in plant-based food products, contributing both to health benefits and potential food preservation through antioxidant activity.

## 1. Introduction

In recent years, there has been a noticeable shift in consumer eating habits, moving away from meat-based products in favor of plant-based alternatives. This change is influenced by factors such as health consciousness and growing environmental awareness [1]. Consumers are becoming aware of the health advantages associated with plant-based diets, which tend to be nutrient-dense and lower in saturated fats when compared with diets that focus on animal products [2]. Studies have also demonstrated that plant-based diets are associated with a reduced incidence of non-communicable diseases, but could still be detrimental to bone health [3]. While the health benefits of plant-based foods have often been attributed to phytochemical compounds such as polyphenols and carotenoids, emerging evidence highlights the important role of plant proteins and their derived peptides in promoting health [4,5].

Oilseed crops have especially gained attention as a source of protein meals, a by-product of the oil extraction process, as they are naturally rich in high-quality proteins. As a result, protein meals traditionally used for livestock feed could serve as a good alternative protein source to meet the increasing demand for plant protein-based foods [6]. Rapeseed (*Brassica napus* L.) is the third-largest oilseed crop globally, with a production of 91 million tons in 2023 (FAOSTAT, 2025). It is the second-leading source of protein meal after soybeans. Rapeseed meal is rich in protein, containing approximately 34% on a dry matter basis, making it a suitable source for producing hydrolysates rich in bioactive peptides [7,8,9]. The protein composition of rapeseed meal mainly consists of two types of storage proteins, which have distinct properties. The 12S globulins, known as cruciferins, are the most abundant fraction, making up 50–70% of the total proteins. They have a molecular weight of about 300 kDa and an isoelectric point of 7.2. In contrast, the 2S albumins, referred to as napins, are low-molecular-weight proteins (10–15 kDa) and account for 20 to 45% of the total rapeseed proteins [10,11]. Rapeseed albumins (RAs) have a compact heterodimeric structure and are soluble across a wide range of pH levels due to a high isoelectric point of 11 [12]. RAs present a well-balanced composition of essential amino acids, particularly being rich in sulfur-containing residues compared with other plant proteins, such as those from soybeans, for example [13]. These characteristics make RA attractive candidates for incorporation into plant-based food formulations, especially for consumers seeking alternative protein sources, such as vegans [10]. However, their compact and disulfide-rich structure also makes them highly resistant to enzymatic hydrolysis, including digestive enzymes such as pepsin, which can limit their digestibility and the release of potentially beneficial peptides. Furthermore, previous studies have demonstrated that peptides derived from rapeseed meal proteins exhibit bioactive properties, including antioxidant and anti-inflammatory effects, which can enhance the health benefits of food products [9,14,15]. These bioactive peptides from RAs are typically released through enzymatic hydrolysis using commercial proteases. However, fermentation by lactic acid bacteria, such as *Streptococcus thermophilus*, offers a natural and potentially consumer-friendly alternative for producing such peptides.

*Streptococcus thermophilus* is the second most important species of industrial dairy starter after *Lactococcus lactis* and is a key ingredient in the production of fermented milk products, particularly yogurt and certain cheeses. One reason for its widespread use in the dairy industry is its recognized safety profile: it is classified as Generally Recognized as Safe (GRAS) by the U.S. Food and Drug Administration (FDA) and has a Qualified Presumption of Safety (QPS) designation from the European Food Safety Authority (EFSA) [16,17]. In addition, *S. thermophilus* displayed various positive health effects, including the prevention of gastritis and infectious diarrhea, as well as the alleviation of lactose intolerance [18]. In 2010, the EFSA approved a health claim for yogurt’s ability to improve lactose digestion in lactose-intolerant individuals, an effect attributed to the presence of *S. thermophilus* (EFSA, 2010). *S. thermophilus* was also observed to significantly upregulate the expression of anti-inflammatory cytokines (IL-4, IL-5, and IL-10), while concurrently reducing the secretion of pro-inflammatory cytokines (IL-1β and IFN-γ) in spleen cells co-cultured with an agonist peptide [19]. Recently, it has been shown that the peptides obtained from the surface proteins of this bacterium displayed anti-inflammatory activity by reducing the secretion of pro-inflammatory cytokines in in vitro cell models [20].

*S. thermophilus* generates secondary products during lactose fermentation that contribute to the aroma and texture of the final product. It also rapidly acidifies milk by converting lactose into lactic acid [21,22]. Additionally, *S. thermophilus* possesses a proteolytic system, which includes cell-wall protease (PrtS), extracellular peptidases, amino acids, and peptide transport systems, as well as a variety of intracellular peptidases [23,24,25]. Although PrtS protease is the primary enzyme in the proteolytic system responsible for hydrolyzing milk proteins during fermentation, it is not present in all strains and is not always anchored to the cell wall [26]. For instance, in the LMD-9 strain, PrtS protease is anchored to the cell wall, while in the 4F44 strain, it exists in both anchored and free forms [27,28]. Studies have demonstrated that both the anchored and free forms of PrtS protease can generate a variety of peptides from milk proteins, which possess diverse bioactivities in addition to providing peptides necessary for the growth of the bacterium [29,30,31]. For example, Chang and coworkers (2014) reported 22 peptides from bovine caseins as being angiotensin-converting enzyme inhibitors, antioxidants, immunomodulating, or antibacterial peptides [30].

The proteolytic system of *S. thermophilus* is well known for its effectiveness on milk proteins, but its application to plant proteins is limited. This limitation can be attributed to a relatively recent interest in plant-based protein substrates, as well as the complex structure of these molecules, particularly the compact structure of RAs, which makes them challenging to hydrolyze. It was, indeed, reported that RAs exhibit high resistance to gastrointestinal hydrolysis, especially to pepsin digestion [32]. However, the use of *S. thermophilus* as a tool for plant protein transformation could be highly beneficial. Indeed, the fermentation of plant proteins by *S. thermophilus* can serve multiple purposes. The proteolytic activity of *S. thermophilus* during fermentation can modify the conformation and accessibility of proteins by hydrolyzing them into low-molecular-weight polypeptides, thereby enhancing their digestibility and unlocking bioactive sequences. Particularly, during the fermentation of soy milk, *S. thermophilus* has been shown to hydrolyze soy proteins that could help to improve the digestibility of soy-based products. This process may also provide additional health benefits by reducing the allergenicity of soy proteins and/or promoting the production of bioactive peptides [33]. Fermentation by *S. thermophilus* can also facilitate the extraction of proteins from plant sources with improved physicochemical properties compared with conventional methods. For instance, lactic acid fermentation involving *S. thermophilus* and/or other lactic acid bacteria has been shown to enhance both the yield and solubility of albumin proteins extracted from peas [34]. Furthermore, fermentation with *S. thermophilus* can align with clean-label trends by enabling natural processing without the need for commercial enzymes or harsh chemicals. Thus, biotransformation using this bacterium could not only improve the functional and nutritional quality of plant proteins but also expand their application in health-oriented, plant-based food formulations, meeting the growing consumer demand for sustainable and naturally processed products.

The primary objective of this study was to investigate the proteolytic capabilities of two strains of *S. thermophilus* in hydrolyzing RA. First, we examined the proteolytic activity of the PrtS protease from the *S. thermophilus* LMD-9 and 4F44 strains. This was followed by a kinetic and mechanistic study of RA hydrolysis using concentrated cell pellets from both *S. thermophilus* strains. Subsequently, we produced two RA hydrolysates using the LMD-9 strain, which were then evaluated for their potential in vitro antioxidant and anti-inflammatory activities in a cell model. These findings contribute to the development of novel plant-based functional ingredients through microbial bioprocessing, advancing sustainable food innovation.

## 2. Materials and Methods

### 2.1. Materials and Chemicals

RAs were obtained from de-oiled rapeseed meal according to the original patented process [35]. It is an isolate with a protein purity of approximately 99% (measured by the Kjeldahl method; Nx6.25, on a dry matter basis) and presents a high solubility (~100%) over a wide pH range (from 3 to 9).

All solvents (water and acetonitrile) were HPLC-grade and were purchased from Fisher Scientific (Hampton, VA, USA). The synthetic peptides used for the column calibration were purchased from GeneCust (Dudelange, Luxembourg). All other chemicals and reagents used were of analytical grade.

### 2.2. Bacterial Strains, Culture, and Growth Conditions

Two *S. thermophilus* strains were used in this study. The *S. thermophilus* LMD-9 (ATCC BAA-491) strain was obtained from the American Type Culture Collection (Manassas, VA, USA), whereas the *S. thermophilus* 4F44 strain was isolated in the laboratory from cheese. Both strains were conserved at −20 °C in skim milk reconstituted at 10% (*w*/*v*). To monitor their growth, each strain was precultured separately at 1% in 10% sterile reconstituted skim milk and incubated overnight at 42 °C. The following day, LM17 broth (M17 supplemented with 20% lactose, with a *v*/*v* ratio of 9:1) [36] was inoculated at 1% with a preculture of each strain and again incubated at 42 °C. The growth was followed by measuring the optical density (OD) at 650 nm every 30 min of each strain culture. During each experiment, LM17 broth and skim milk medium were incubated alone under similar conditions as a negative control.

### 2.3. Specific Activity of Cell-Wall Protease PrtS

To measure the specific activity of the PrtS protease, both strains were first precultured and cultured in LM17 broth as described above. The OD at 650 nm of each strain culture was monitored regularly, and the samples were taken at different OD_650nm_ values (1 to 5). The aliquoted samples were centrifuged at 3220× *g* for 15 min at 20 °C, and the obtained cell pellets were resuspended in sterile 100 mM Tris-HCl buffer (pH 7.5) containing 5 mM CaCl_2_ and centrifuged under similar conditions. This washing step was repeated 2 times. Then, the specific activity of the PrtS protease was evaluated using a chromogenic substrate, Suc-Ala-Ala-Pro-Phe-*p*Na (Sigma-Aldrich, Saint Quentin Fallavier, France), as described previously by [28]. Briefly, a synthetic substrate (1.24 g/L) was prepared in 1 vol of N, N-dimethyl formamide and 9 vol of 50 mM Tris-HCl buffer (pH 8), supplemented with 5 mM CaCl_2_. The bacterial pellets corresponding to 5 mL of the cultures obtained at different OD_650nm_ values were resuspended in 5 mL of this chromogenic substrate solution. The mixture was incubated at 37 °C with stirring at 180 rpm, and samples were drawn at different reaction times, i.e., 15 min, 30 min, 45 min, 60 min, 90 min, 120 min, 180 min, and 240 min. The samples were centrifuged at 16,000× *g* for 1 min at 4 °C, and the absorbance was measured at 410 nm. The amount of *p*-nitroaniline liberated during the enzymatic reaction was calculated using an extinction coefficient of 8800 M^−1^.cm^−1^ [37]. The specific activity was expressed as µmol of the *p*Na released per unit time per OD under the experimental conditions. The experiment was repeated three times for each strain. The control was made under similar conditions but without any bacterial pellet.

### 2.4. Hydrolysis of Rapeseed Albumins by PrtS Protease

The RA solution at a concentration of 0.1% was prepared by dissolving RA powder in 100 mM Tris-HCl buffer (pH 7.5) supplemented with 5 mM CaCl_2_. Subsequently, it was filtered through a sterile filter with a porosity of 0.2 μm (Pall corporation, Saint Germain en Laye, France) and stored at 4 °C for later use.

To study RA hydrolysis by *S. thermophilus* strains, both LMD-9 and 4F44 strains were first propagated in LM17 broth up to the exponential growth phase (OD_650nm_ of 1 ± 0.05), and the bacterial cells were harvested and washed twice as described for PrtS specific activity. Once washed, the cells were resuspended in 100 mM Tris-HCl buffer (pH 7.5) and supplemented with 5 mM CaCl_2_ to achieve cell concentrations of 10X and 100X. Indeed, the varying cell concentrations corresponded to varying PrtS protease concentrations. Then, these cell suspensions were, again, centrifuged under similar conditions, and the obtained cell pellets were incubated with a 0.1% RA solution at 37 °C with stirring (180 rpm). In parallel, the RA solution was incubated alone in the same conditions to verify its stability. Samples were drawn at 0 h, 1 h, 2 h, 4 h, and 24 h of incubation and centrifuged at 16,000× *g* for 1 min at 4 °C to obtain the supernatant corresponding to the RA hydrolysate. Then, 5 µL of 5 M HCl was added to 1 mL of the RA hydrolysate to stop the enzymatic reactions. The medium following the addition of HCl became cloudy, and the final centrifugation of the hydrolysate was carried out under the same conditions, but for 5 min, and it was filtered through a filter with a porosity of 0.45 μm (Phenomenex, Le Pecq, France) and stored at −20 °C until further analysis.

### 2.5. Characterization of Hydrolysates by Size-Exclusion High-Performance Liquid Chromatography (SE-HPLC) Analysis

RA hydrolysates recovered at different times were analyzed and characterized by SE-HPLC according to Beaubier et al. [38]. Briefly, 25 µL of the RA hydrolysates was loaded into a Superdex peptide 10/300 GL column (10 × 300 mm, Cytiva, MA, USA) connected to a Shimadzu model LC20 system (Shimadzu Corporation, Japan). The column temperature was maintained at 35 °C. The samples were eluted for 60 min using isocratic elution with water containing 30% acetonitrile and 0.1% trifluoroacetic acid (69.9:30:0.1, *v*/*v*) at a flow rate of 0.5 mL/min. The elution of the samples was monitored using a UV signal at 214 nm with a cell having an optical path of 1 cm. The column calibration was performed with synthetic peptides (GeneCust, Dudelange, Luxembourg) of known molecular masses, which were eluted under similar conditions. The data were processed by the Labsolution software (Version 5.87 SP1, Shimadzu Corporation, Kyoto, Japan) and then exported into Excel spreadsheets for the quantification of parameters related to hydrolysis kinetics.

For the characterization of RA hydrolysates, three protein hydrolysis parameters, i.e., the protein conversion rate (Xp), the average number of amino acid residues per peptide (Naa), and the degree of hydrolysis (DH), were determined according to Beaubier et al. [38]. This method is based on determining the molar extinction coefficient at 214 nm of each “x” point of the chromatogram to convert the absorbance profiles into concentration profiles using the Beer–Lambert law. The DH represents the percentage of cleaved peptide bonds compared with the initial number of peptide bonds in the protein, and Xp is the proportion of protein hydrolyzed at a given time of the reaction progression. Naa represents the mean size of the peptides produced.

### 2.6. Evaluation of Bioactivities

#### 2.6.1. Anti-Inflammatory Activity

The anti-inflammatory activity of the RA hydrolysates was evaluated using the human adenocarcinoma HT-29 cell line (ECACC, Sigma-Aldrich, Saint Quentin Fallavier, France), as described by Allouche et al. [20]. Shortly, HT-29 cells were maintained in McCoy’s 5A Medium (Gibco, Villebon-sur-Yvette, France) supplemented with 10% heat-inactivated fetal bovine serum (FBS) and a 1% streptomycin/penicillin antibiotic solution at 37 °C in a humidified 5% CO_2_ atmosphere. The confluent cells were washed and trypsinized with 0.25% trypsin–EDTA 1X. Then, the trypsinized cells, at a concentration of 1 × 10^5^ cells/mL in McCoy’s 5A medium, were seeded (500 µL per well) in a 24-well plate and again incubated under similar conditions. The medium was changed every 24 h for 4 days; however, the FBS concentration was reduced to 5% in McCoy’s 5A medium on the 4th day and during co-treatment. During co-treatment, the cells in each well were fed with 450 µL of McCoy’s 5A medium containing 5% FBS without antibiotics. Then, 50 µL of either RA hydrolysates (5 and 10 mg/mL) with 50 ng/mL of lipopolysaccharide (LPS) from *Escherichia coli* O111: B4 (Sigma-Aldrich, Saint Quentin Fallavier, France) or LPS alone or the medium were added to the cells. Untreated cells or those treated with LPS alone served as negative and positive controls, respectively. Following 3 h of incubation in the same conditions, the supernatants were collected by centrifugation at 14,000× *g* for 5 min at 4 °C and conserved at −80 °C until further analysis.

The cells were used to conduct a cytotoxicity test to determine whether the RA hydrolysates were cytotoxic for the cells at the tested concentrations and for a given period of co-incubation. Following the twice washing of the cells with PBS 1X (Gibco™), 500 μL of a 0.05% Crystal Violet dye (Sigma Aldrich, Saint Quentin Fallavier, France) solution (prepared in 10% ethanol) was added to each well and incubated for 20 min at room temperature. Then, the crystal violet solution was removed, and the cells were washed thrice with the same buffer. After air drying the cells, the crystal violet dye retained by alive cells was dissolved by adding 500 μL of 10% acetic acid to each well, which was quantified by measuring the absorbance at 590 nm using a microplate spectrophotometer (Epoch™ 2, BioTek® Instruments, Winooski, VT, USA). The percentage of cell viability was calculated using the following equation:

%Cell viability = [(absorbance of treated cells)/(absorbance of untreated cells] × 100

The pro-inflammatory cytokine IL-8 released in the supernatants was quantified using ELISA kits (Thermo Fisher Scientific, Villebon-sur-Yvette, France) following the manufacturer’s instructions. The experiments were repeated three times, and every time, two technical replicates were performed.

#### 2.6.2. ABTS Radical-Scavenging Activity

The antiradical activity of RA and its hydrolysates was determined according to a previously described method by Sadat et al. [39], with modifications. Firstly, ABTS (acid 2,2’-azinobis [3-éthylbenzothiazoline-6-sulfonique]) cation radical (ABTS^•+^) was prepared by oxidizing 7 mM ABTS molecules (Sigma-Aldrich, Saint Quentin Fallavier, France) with 2.45 mM potassium persulfate (final concentration) for 12–16 h at room temperature in the absence of light. Then, the initial absorbance at 740 nm of the ABTS^•+^ solution was adjusted to 0.65–0.70 by diluting (1/80) the former in 5 mM sodium phosphate buffer (pH 7.4) (SPB). The radical species was stable in this solution for 1 h at 22 °C. Stock solutions of RA and its hydrolysates were prepared in SPB, while those of Trolox, an analog of vitamin E (Sigma-Aldrich, Saint Quentin Fallavier, France), and gallic acid (Sigma-Aldrich, Saint Quentin Fallavier, France) were prepared in ethanol and SPB, respectively. The initial concentrations of RA, RA hydrolysates (0–100 mg/mL), Trolox (0–100 µM), and gallic acid (0–40 µM) were prepared by diluting stock solutions with SPB to allow the gradual reduction of the ABTS^•+^. The reaction was initiated by mixing 30 µL of the sample or a standard with 270 µL of diluted ABTS^•+^ in a microplate that was incubated in the dark at 20 °C for 10 min. The decrease in absorbance was measured at 740 nm using a microplate spectrophotometer. All the measurements were performed at least three times and in triplicate. The ABTS scavenging activity was expressed in % and was calculated as follows:

% anti-radical activity = [(A_remaining_ − A_blank_)/(A_ABTSinitial_ − A_blank_)] × 100

where A_blank_ corresponds to 300 µL of SPB, and ABTS initial absorbance corresponds to 270 µL of ABTS^•+^ and 30 µL of SPB. The ABTS radical-scavenging activities were calculated as mM Trolox equivalents per g of the sample.

### 2.7. Statistical Analysis

The results of ELISA were expressed as means ± the standard errors of the means (SEMs) from three independent experiments. The data were analyzed using the Kruskal–Wallis test for comparisons between multiple groups and the Mann–Whitney U test for pairwise comparisons. All statistical analyses were carried out with GraphPad Prism 10.4.1 (GraphPad Software, San Diego, CA, USA). A *p*-value of <0.05 was considered statistically significant.

## 3. Results and Discussion

### 3.1. Proteolytic Activity of PrtS Protease from S. thermophilus LMD-9 and S. thermophilus 4F44 Strains

**Figure 1:**
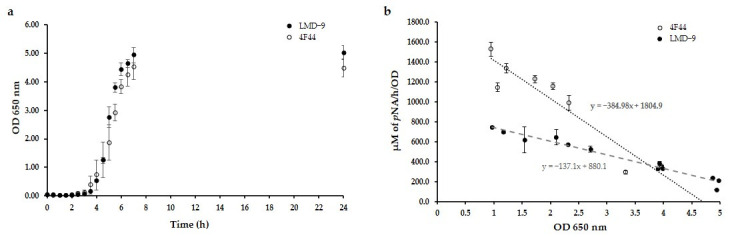
Growth in LM17 broth (**a**) and specific activity of PrtS protease (**b**) of *Streptococcus thermophilus* LMD-9 (●) and 4F44 (○) using Suc-Ala-Ala-Pro-Phe-Lys-*p*NA as substrate. The synthetic substrate was prepared at 1.24 g/L in 1 vol of N, N-dimethyl formamide and 9 vol of 50 mM Tris HCl buffer (pH 8), supplemented with 5 mM CaCl_2_. The specific activity was defined as µmol of the *p*Na released per unit time per OD under experimental conditions. Results are presented as means of 3 observations.

In this study, two strains of *S. thermophilus* were investigated. The LMD-9 strain, in which PrtS protease was anchored to the cell wall, and the 4F44 strain, in which approximately 60% of the PrtS protease was attached to the wall of the bacteria, and about 40% was secreted in the growth medium due to an anchoring defect of the SrtA sortase [28]. First, the growth curves of both strains were established in LM17 broth to identify their different growth phases. As shown in Figure 1a, the LMD-9 and 4F44 strains entered the exponential growth phase almost at the same time, i.e., after 210 min. Samples drawn at different OD_650nm_ values represent the start to end of the exponential growth phase and the start of the stationary phase. Indeed, Galia and co-workers (2016) reported that PrtS protease displayed maximal activity during the exponential growth phase in LM17 broth [40].

Hence, the samples drawn at different phases of the exponential growth phase and the start of the stationary phase of the *S. thermophilus* LMD-9 and 4F44 strains were used to determine the specific activity of PrtS protease using the specific synthetic substrate Suc-Ala-Ala-Pro-Phe-*p*Na. The specific activity was expressed as µM of *p*NA released by the action of PrtS protease from this substrate per hour and per unit of OD_650nm_ and is presented in Figure 1b. The PrtS protease of the LMD-9 strain displayed a decrease in the specific activity with increasing OD_650nm_, ranging from 773.8 µM of *p*NA/h/OD for an OD_650nm_ of 1 (corresponding to the initial exponential phase) to 224.9 µM of *p*NA/h/OD for an OD_650nm_ of approximately 5 (corresponding to the start of the stationary phase). The same phenomenon was observed for PrtS protease of the 4F44 strain, with values ranging from 1635.5 µM of *p*NA/h/OD to 391.6 µM of *p*NA/h/OD for OD_650nm_ of 1 to approximately 4. The results are consistent with the previous findings, where the authors reported a decrease in PrtS protease activity with increasing OD_650nm_ values [28,40]. Moreover, the specific activity of the PrtS protease of the 4F44 strain was approximately twice as high as that of the LMD-9 strain at an OD_650nm_ value equal to 1, i.e., the start of the exponential phase. This was surprising since a higher specific activity for the LMD-9 strain could have been expected since the 4F44 strain loses some of the PrtS proteases in LM17 broth. The 4F44 strain may compensate for the loss of the protease by a higher expression of its gene, which would result in a higher specific activity.

However, the specific activity of both strains was similar at an OD_650nm_ equal to 4. The decrease in specific activity as a function of the OD_650nm_ was 3 times faster in the 4F44 strain than in the LMD-9 strain. Keeping in view these observations of the specificity of both *S. thermophilus* strains, an OD_650nm_ value of 1, corresponding to the start of the exponential phase, was chosen to subsequently carry out the RA hydrolyses with concentrated cell pellets of both strains.

### 3.2. Study of Rapeseed Albumin Hydrolysis Using Concentrated Cell Pellets of S. thermophilus 4F44 and LMD-9 Strains

#### 3.2.1. Hydrolysis Kinetics Study Using Size-Exclusion Chromatography Analysis

**Figure 2:**
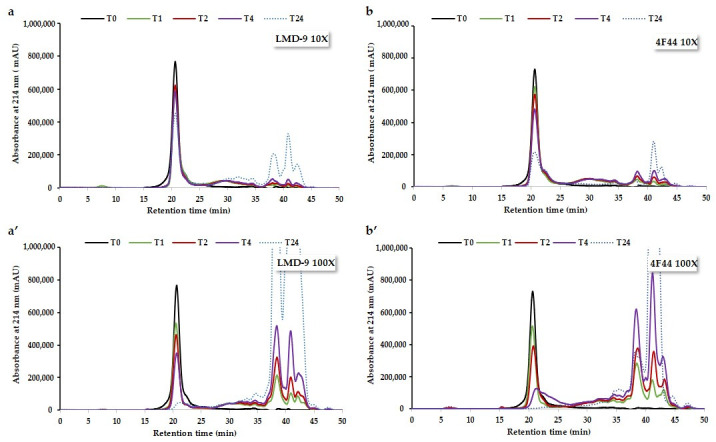
Size-exclusion chromatograms at 214 nm of samples drawn during hydrolysis of rapeseed albumins by the PrtS protease from cells concentrated 10X (**a**,**b**) and 100X (**a’**,**b’**) of *S. thermophilus* LMD-9 (**a**,**a’**) and 4F44 (**b**,**b’**) strains. Both strains were grown to an OD_650nm_ of 1 in LM17 broth. Followed by washing with 100 mM Tris-HCl buffer (pH 7.5), supplemented with 5 mM CaCl_2_. The cells of each strain were concentrated 10x and 100x to increase the PrtS protease quantity and incubated with a 0,1% rapeseed albumin solution for up to 24 h at 37 °C. Samples were drawn at the beginning (T0) and after 1 (T1), 2 (T2), 4 (T4), and 24 h (T24) of hydrolysis.

The hydrolysis of RAs by the PrtS protease of concentrated cells (10X and 100X) of the *S. thermophilus* LMD-9 and 4F44 strains was conducted for 1 to 24 h, with samples taken at various time intervals and analyzed using SE-HPLC. The chromatograms obtained at 214 nm are presented in Figure 2. They revealed that the unhydrolyzed RAs (12–15 kDa) were eluted at 20 min, as previously described by Beaubier et al. [38]. The peptides resulting from these hydrolyses were eluted between 23 and 35.5 min (i.e., from 2300 g/mol to 300 g/mol), regardless of the bacterial strain. The UV signals observed after 35.5 min were attributed to nucleic acids. Indeed, the detection at 260 nm of the hydrolysates highlighted bacterial lysis, leading to the release of nucleic acids, eluted from 35.5 min.

For all the hydrolysis performed, regardless of the parameters, the RA signal disappeared, while the peptide signals increased as the hydrolysis progressed. Hence, it can initially be stated that the PrtS protease of the *S. thermophilus* LMD-9 and 4F44 strains can hydrolyze RA. This finding is noteworthy because RA can be resistant to proteolysis due to its structural stability, explained by the presence of four disulfide bridges [12], which may limit the accessibility of the protease to peptide bonds [32].

**Figure 3:**
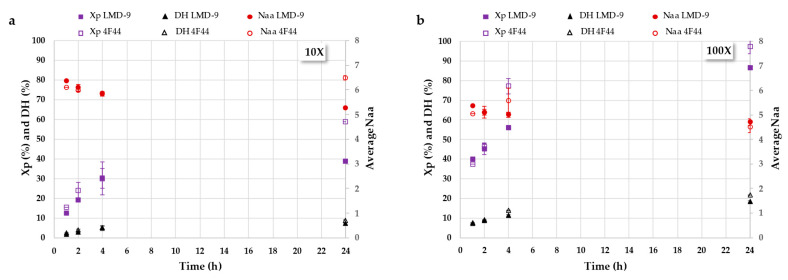
Hydrolysis kinetics, i.e., protein conversion rates (Xp; %, squares), degrees of hydrolysis (DH; %, triangles), and mean numbers of residues per peptide (Naa; round) for the hydrolysis of rapeseed albumins with PrtS protease from *S. thermophilus* strains (filled marks: LMD9; empty marks: 4F44) over 24 h for both cell concentrations 10X (**a**) and 100X (**b**).

Analyzing the chromatograms alone is insufficient for comparing the hydrolysis efficiency of the proteases from the LMD-9 and 4F44 strains. To facilitate a more accurate comparison, it is crucial to determine the kinetic parameters, including the protein conversion rate (Xp), which represents the percentage of hydrolyzed protein; the degree of hydrolysis (DH), indicating the number of peptide bonds cleaved; and the average number of residues per peptide (Naa), reflecting the mean size of peptides produced in the hydrolysates [38]. Figure 3 shows the kinetics of these parameters monitored during the hydrolysis of RA by the PrtS protease of concentrated 10X (Figure 3a) and 100X (Figure 3b) cells of the *S. thermophilus* LMD-9 and 4F44 strains.

When RAs were incubated with 10-fold-concentrated cells, moderate hydrolysis occurred over 24 h, with the 4F44 strain showing a conversion rate ranging from 15% (after 1 h) to 59%, and the LMD-9 strain ranging from 12% (after 1 h) to 39%. This indicates that more than 40% of the RAs initially added to the hydrolysis reaction remained unhydrolyzed after 24 h. This could be explained by the insufficient quantity of proteases at the applied cell concentration, protease deactivation, or limited accessibility of certain protein cleavage sites under the given conditions. The enzyme/substrate ratio (E/S) is a major operating condition in proteolysis and plays an important role in kinetic studies [41]. Moreover, a high protein concentration may lead to enzymatic deactivation [42]. Under these conditions, maximum DH values of 9%, considered as so-called limited hydrolysis [41], were obtained.

A 10-fold higher cell concentration (i.e., 100X), and, thus, a higher PrtS protease concentration, was studied to assess its impact on hydrolysis kinetics. As shown in Figure 3b, the RA hydrolysis rate was significantly higher by a factor of three. Thus, 40% of the initial RA was depleted after just 1 h of contact, and nearly 100% was hydrolyzed by the end of the 24 h, resulting in the production of peptides and free amino acids. Therefore, this cell concentration provided a sufficient amount of PrtS protease to hydrolyze RA at the studied concentration of 0.1% w/v. These observations were quite similar for both studied *S. thermophilus* strains. However, the presence of nucleic acids in the hydrolysates (Figure 2; UV signal at 260 nm) indicates that cell lysis occurred, potentially leading to the release of intracellular peptidases involved in RA hydrolysis. Nevertheless, the contribution of these intracellular peptidases was likely limited, as previous studies have shown that the PrtS protease predominates in the proteolysis reaction when it is present [25]. Given the concentrations used, DH values of approximately 20% were reached after 24 h, indicating extensive hydrolysis and resulting in the formation of small peptides, averaging at around five residues, findings that are particularly relevant for the potential release of bioactive peptides, which typically consist of 2 to 20 residues [43] with no residual RA, known for its poor digestibility [32].

#### 3.2.2. Deciphering Proteolysis Mechanism of Rapeseed Albumins by *S. thermophilus* PrtS Protease of Studied Strains

As highlighted in a previous study [44], relying solely on DH kinetics is insufficient to effectively control the proteolysis process required for producing functional or bioactive hydrolysates. To achieve this level of control, it is essential to identify the underlying mechanism(s), as this enables the production of hydrolysates with targeted characteristics, particularly in terms of the residual protein content (Xp) and peptide size (Naa).

According to the Linderstrøm-Lang theory, proteolysis can be described by two main mechanisms: the “zipper” mechanism and the “one-by-one” mechanism. These models differ based on the accessibility of native proteins to enzymatic action and the relative rates of the key steps involved in proteolysis [41,45]. The process is generally divided into two stages: (i) the initial denaturation of the protein, which unmasks peptide bonds (*k*_1_), and (ii) the subsequent hydrolysis of these exposed peptide bonds (*k*_2_). In the “one-by-one” mechanism, the initial denaturation step is the rate-limiting factor (*k*_1_ *< k*_2_). Proteins are hydrolyzed sequentially, with each molecule being fully broken down before the next is processed. As a result, no intermediate-sized peptides are observed, and the peptide profile remains relatively stable over time. Conversely, the “zipper” mechanism features a rapid denaturing and unfolding phase (*k*_1_ *> k*_2_), allowing all protein molecules to become simultaneously accessible to enzymatic cleavage. This leads to the generation of a broad spectrum of intermediate peptides of various sizes throughout the reaction. Consequently, the characteristics of the resulting hydrolysates can be linked to the mechanism at play: the “one-by-one” model typically yields a mixture of residual intact proteins and small peptides, while the “zipper” mechanism produces a diverse array of peptides with a wide range of molecular sizes [41].

**Figure 4:**
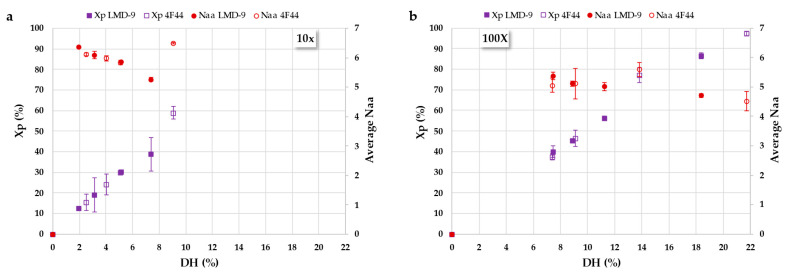
Protein conversion rate (Xp; %, squares) and mean number of residues per peptide (Naa; circles) for the hydrolysis of rapeseed albumins by PrtS protease from *S. thermophilus* strains (filled markers for LMD-9 and empty markers for 4F44), as a function of degree of hydrolysis (DH, %), determined by size-exclusion chromatography, at cell concentrations of 10X (**a**) and 100X (**b**).

Figure 4 presents the protein conversion rate (Xp) and the average number of residues per peptide (Naa) plotted against the degree of hydrolysis (DH) for both *S. thermophilus* strains (LMD-9 and 4F44) at two cell concentrations (10X and 100X). These plots provide insight into the hydrolysis mechanism [38,46]. Regardless of the cell concentration used, a linear relationship was observed between the conversion rate and DH, consistent across the PrtS proteases of both *S. thermophilus* strains. This indicates that at a given DH value, a similar protein conversion rate can be achieved independent of the strain or cell concentration. However, the time required to reach this rate varies between strains and concentrations, suggesting that while the strain and enzyme-to-substrate (E/S) ratio influence the kinetics of hydrolysis, they do not affect the underlying mechanism. Consequently, the composition of the hydrolysate—i.e., the residual proteins and peptide size—remains unchanged. While the kinetic impact of the E/S ratio has been previously described [42,44], the similar proteolytic behavior of the PrtS proteases from the two different strains with RA has not been reported before. In this case study, a single hydrolysis mechanism was apparent, characterized by a linear trend in Naa, which remained relatively stable at approximately 5, regardless of the DH. This suggests that proteolysis proceeds sequentially, consistent with a preferential “one-by-one” cleavage mechanism.

### 3.3. Characterization of Bioactivities of Rapeseed Albumin Hydrolysates Generated by PrtS Protease from S. thermophilus

#### 3.3.1. Production of Rapeseed Albumin Hydrolysates Using Concentrated Cell Pellets of *S. thermophilus* LMD-9 Strain

In the continuation of this study, we aimed to produce RA hydrolysates using PrtS protease to evaluate their potential anti-inflammatory and antioxidant bioactivities. The previous findings indicated that the PrtS proteases from two *S. thermophilus* strains exhibited similar proteolytic behavior with RA. Therefore, we chose to use only the LMD-9 strain for hydrolysate production. This strain was particularly suitable as its PrtS protease is anchored to the cell wall and is already utilized in the production of fermented dairy products. The identified hydrolysis mechanism follows a “one-by-one” pattern, where the amount of residual protein decreases as the reaction progresses. Since a higher cell concentration enhances reaction progression, we opted for a 100X cell concentration for hydrolysate production. To assess potential variations, we scaled up the production and generated two hydrolysates with hydrolysis durations of 4 h (H4) and 24 h (H24).

Both hydrolysates were characterized using SE-HPLC. Hydrolysate H4 exhibited a DH of 11.2%, with 44% residual RA (Xp of 56%) and peptides averaging at five residues. In contrast, hydrolysate H24 had a DH of 18.4%, with 13% residual RA (Xp of 87%) and peptides averaging at 4.7 residues. The primary difference between the two hydrolysates was the amount of residual proteins, which was three times higher in H4 than in H24.

#### 3.3.2. Antioxidant Activity of Rapeseed Albumin Hydrolysates

**Figure 5:**
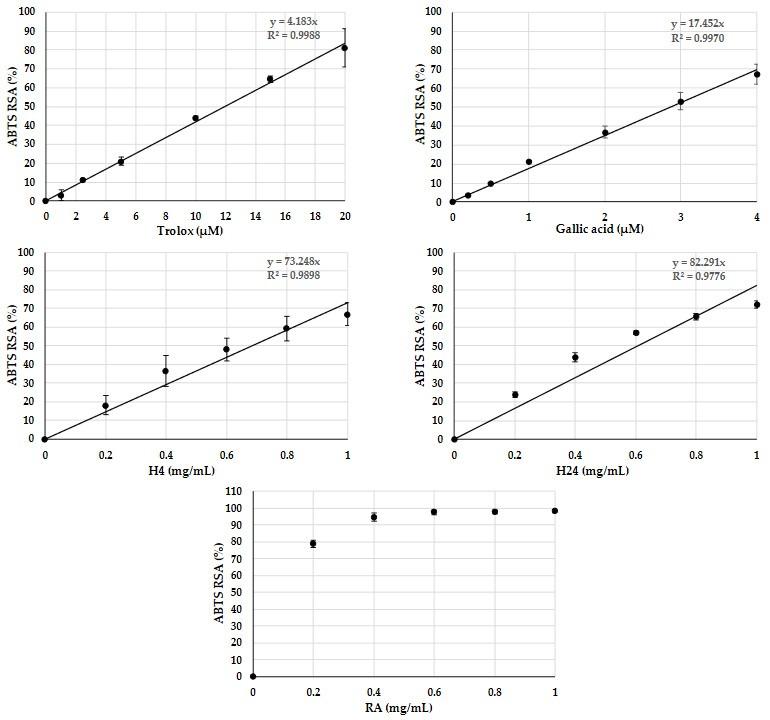
Relationship between concentration and radical-scavenging activity of antioxidants Trolox and gallic acid, and rapeseed albumins (RAs) and hydrolysates of these proteins (H4 and H24), which were generated by PrtS protease from *S. thermophilus* LMD-9 strain after 4h or 24h. The activity was measured by the ABTS assay at 740 nm using a microplate reader. Experiments were performed in triplicate. Error bars show standard deviations. ABTS RSA: ABTS radical-scavenging activity.

ABTS is a stable organic free radical that directly measures a sample’s scavenging ability by monitoring changes in absorbance. The ABTS radical-scavenging activities (in %) and IC_50_ values of RA, H4, and H24 hydrolysates were determined across a range of concentrations and compared with those of the well-known synthetic antioxidants Trolox and gallic acid. The resulting antiradical activities are presented in Figure 5.

A linear relationship was observed between the concentration and response in the range 0 to 20 μM for Trolox and 0 to 4 μM for gallic acid. The IC_50_ values derived from the concentration–response curves for gallic acid and Trolox were 2.88 ± 0.21 μM (0.49 ± 0.04 mg/L) and 11.98 ± 0.78 μM (3.00 ± 0.20 mg/L), respectively. The IC_50_ value for gallic acid was similar to that reported by Sadat et al. [39]. At 4 μM, gallic acid exhibited an ABTS radical-scavenging activity of approximately 67%, while Trolox at 20 μM showed 81% activity. Non-hydrolyzed RA demonstrated an antioxidant activity of 78% at a concentration of 0.2 mg/mL, which increased to 95% at 0.4 mg/mL. This activity remained constant between 0.4 and 2 mg/mL, likely due to the saturation of RA from a concentration of 0.4 mg/mL. The IC_50_ value of the RA was determined to be 8.46 ± 0.03 μM (considering a molar mass of 15 kDa for RA), which was noteworthy for this resource and fell between the IC_50_ values of the reference antioxidants, gallic acid, and Trolox. The ABTS radical was scavenged by 67% and 72% at a concentration of 1 mg/mL for the H4 and H24 hydrolysates, respectively. The IC_50_ values of the H4 and H24 hydrolysates were 0.69 ± 0.09 mg/mL (i.e., 0.017 mmol Trolox/g) and 0.61 ± 0.02 mg/mL (i.e., 0.02 mmol Trolox/g), respectively. Due to the complexity of these peptide mixtures, it is challenging to determine their molar mass and, therefore, express their IC_50_ in molar terms. However, both hydrolysates exhibited significantly comparable and moderate antioxidant effects when compared with controls (in mass concentrations). Hence, RA and both hydrolysates can reduce oxidant species in a dose-dependent manner. These results confirm previous findings with RA [9]. According to Zhang et al. [47], Tyr and Trp residues are particularly effective in trapping radicals in the ABTS assay. The frequency of Tyr residues in eukaryotic proteins is approximately 3.1%, while that of Trp residues is around 1.1% [48]. In napin I obtained by neutral extraction, the tyrosine content was 1.7%, and tryptophan accounted for 1.0% [49]. Thus, napins do not contain a significantly different frequency of tryptophan residues compared with the average in eukaryotic proteins, and their tyrosine content is lower. Unless aromatic residues are located close and end up in the same peptide, the likelihood of generating peptides rich in aromatic residues remains low. Therefore, the antioxidant activity is likely also attributable to the presence of other amino acid residues.

#### 3.3.3. Anti-Inflammatory Activity of Rapeseed Albumin Hydrolysates

The anti-inflammatory activities of RA and its hydrolysates (H4 and H24) were assessed in vitro using the human colorectal adenocarcinoma HT-29 cell line. This cell line is commonly used to study the bioavailability or inflammatory response of food components, in addition to its conventional application in colon cancer research. It mimics the characteristics of mature intestinal epithelial cells [50,51], which are continuously exposed to food components and various harmful stimuli that can induce an immune response in the epithelium [52]. Under pro-inflammatory conditions, epithelial cells produce the pro-inflammatory cytokine IL-8, which initiates the inflammatory cascade by recruiting and activating neutrophils and extracellular adhesion molecules [53]. In this study, HT-29 cells were treated for 3 h with an inflammatory activator, LPS at 50 ng/mL, either alone or in combination with different concentrations of RA and RA hydrolysates (5.0 and 10.0 mg/mL).

**Figure 6:**
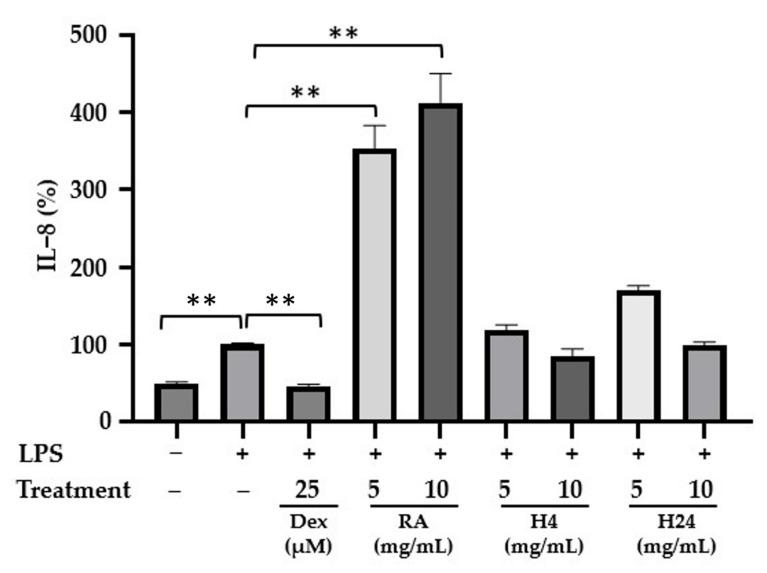
Impact of rapeseed albumins (RAs) and their hydrolysates (H4 and H24) on IL-8 secretion in LPS-stimulated HT-29 cells. HT-29 cells were incubated for 3 h with LPS (50 ng/mL) and with RA or its hydrolysates. IL-8 secretion in the culture medium was measured by ELISA after 3 h of treatment. The negative control (LPS−) represents untreated cells, and dexamethasone (Dex) was used as a positive control at 25 µM. IL-8 secretion, IL-8 (%), is expressed as a percentage of the IL-8 released by cells compared with its release by cells treated with LPS alone. Data are presented as the means ± SEMs of three independent experiments (*n* = 3). ** *p* < 0.01.

Next, the secretion of pro-inflammatory cytokine IL-8 was quantified by ELISA. As shown in Figure 6, LPS-induced cells secreted significantly higher IL-8 levels compared with non-induced cells, with the LPS-induced levels normalized to 100% as a reference. Contrariwise, dexamethasone, a synthetic glucocorticoid, significantly reduced LPS-induced IL-8 production to 46.5% (*p* < 0.01). When the cells were treated with LPS in the presence of RA or its hydrolysates, different responses were observed in terms of IL-8 secretion levels. A significant increase in IL-8 secretion was observed in cells in the presence of RA, regardless of the concentration tested. However, in cells treated with RA hydrolysates (H4 and H24) at 5 and 10 mg/mL, IL-8 secretion was similar to that observed in cells treated with LPS alone. These concentrations of RA hydrolysates were chosen for this study, as no significant difference in IL-8 secretion was observed at concentrations of hydrolysates below 5 mg/mL in a previous study with this cell line [20].

Regarding cytotoxicity, no effect was observed at any of the tested concentrations of LPS, dexamethasone, RA, or its hydrolysates, as the overall cell viability after 3 h of exposure to these compounds remained above 95%.

Recent studies have shown that a napin hydrolysate (SwissProt 17333) exhibits anti-inflammatory activity in RAW264.7 macrophage cells by downregulating the expression of intracellular inflammatory factors [54]. Furthermore, a dipeptide purified from the hydrolysate, which the authors suggest corresponds to the Thr-Leu sequence yet is exclusively found within the protein’s signal peptide, also revealed anti-inflammatory effects in a Caco-2/RAW264.7 co-culture model by reducing the production of NO and PGE2 and the expression of iNOS and COX-2 [54]. In addition, a recent study revealed that administering fermented rapeseed meal to weaned rats reduced the inflammatory response by lowering pro-inflammatory IL-6 secretion in the serum and jejunum tissues [55]. Likewise, Wang et al. [56] demonstrated that fermented soybean meal intake downregulated pro-inflammatory cytokine expression in weaned piglets, providing relief from inflammatory injury. Similarly, sourdough fermented with selected lactic acid bacteria exhibited high antioxidant and anti-inflammatory activities [57].

In this study, RA hydrolysates seemed not to exhibit anti-inflammatory activity, as IL-8 secretion levels in LPS-inflamed cells treated with the hydrolysates were comparable to those observed in cells treated with LPS alone. In contrast, the hydrolysates did not induce the pronounced pro-inflammatory response observed with intact RA in LPS-inflamed cells. The proteolysis of RA by the PrtS protease from the *S. thermophilus* strain LMD-9 effectively eliminated the pro-inflammatory potential of these proteins. Given that the H4 hydrolysate contains 44% unhydrolyzed RA and the H24 hydrolysate contains 13%, it is possible that anti-inflammatory peptides could be present and counteract the pro-inflammatory effects of the intact proteins. Further investigation into the removal of intact proteins from the hydrolysates and the evaluation of these purified hydrolysates in HT-29 cells may provide additional insights.

## 4. Conclusions

In conclusion, *S. thermophilus* strains, through the action of the cell-wall-anchored PrtS protease, are capable of hydrolyzing rapeseed albumins (RAs) via a preferential “one-by-one” mechanism, although a significant enzyme concentration is required. Similar to RA, the RA hydrolysates demonstrated antioxidant activity in vitro. However, in contrast with RA, the RA hydrolysates did not induce an increase in IL-8 secretion in LPS-inflamed cells, and certain peptides within the hydrolysate may counteract the pro-inflammatory effects of non-hydrolyzed albumins still present. These findings suggest that the controlled enzymatic hydrolysis of RA using PrtS protease can produce hydrolysates that mitigate or even negate the pro-inflammatory effect of RA while preserving its beneficial antioxidant properties. Beyond their promising bioactivity, such hydrolysates may serve as functional ingredients in plant-based foods, with potential applications in the development of anti-inflammatory or antioxidant-enriched products. The antioxidant properties of the hydrolysates may also contribute to food preservation by limiting oxidative degradation, offering potential benefits both for consumer health and food shelf-life. Further research is needed to assess their acceptability, digestibility, sensory characteristics, and in vivo efficacy to support their integration into biofunctional food products.

## Data Availability

No new data were created or analyzed in this study. Data sharing is not applicable to this article.
